# Glucocorticoids inhibit the innate immune system of human corneal fibroblast through their suppression of toll-like receptors

**Published:** 2009-11-20

**Authors:** Xiuming Jin, Qin Qin, Lili Tu, Jia Qu

**Affiliations:** 1 Eye Center, Affiliated Second Hospital, School of Medicine, Zhejiang University, Hangzhou, P.R. China; 2 School of Ophthalmology and Optometry, Eye Hospital, Wenzhou Medical College, Wenzhou, P.R. China

## Abstract

**Purpose:**

To evaluate the effect of glucocorticoids on the expression and function of Toll-like receptors (TLRs) in human corneal fibroblasts (HCFs).

**Methods:**

Cultured HCF cells were stimulated with three different concentrations of hydrocortisone. The effect on the expression of *TLR2* and *TLR4* was determined by real-time PCR. The TLR2, TLR4, and pIκB-α proteins were compared by western blot. The release of IL-6 and IL-8 was measured using enzyme-linked immunosorbent assay in the presence and absence of TLR2 and TLR4-specific blocking antibodies.

**Results:**

Incubation of HCFs with hydrocortisone markedly inhibited the expression of *TLR2* and *TLR4* mRNAs and decreased the release of IL-6 and IL-8 in a dose-dependent manner. Western blot analysis confirmed that expression of TLR2, TLR4, and pIκB-α was also downregulated in response to hydrocortisone. The result of ELISA also showed the release of IL-6 and IL-8 can also be inhibited by hydrocortisone. However, all these inhibitions were counteracted after pretreatment with anti-TLR2 and anti-TLR4 monoclonal antibodies.

**Conclusions:**

Glucocorticoids, such as hydrocortisone, can inhibit the expression of TLR2 and TLR4 on HCFs, and thus may increase susceptibility to cornea infections. Our results suggest that topical glucocorticoids may affect the cornea’s innate immunity through TLRs.

## Introduction

The corneal innate immune system consists of multiple cell types. The first layer of defense is the corneal epithelium. Immediately beneath this layer of epithelial cells is the stromal layer (fibroblasts are the principal cellular component), followed by an innermost single layer of endothelial cells. Corneal fibroblasts probably contribute to the local accumulation and activation of leukocytes in the cornea, and play an important role in infectious inflammation [1,2]. Recently, Toll-like receptors (TLRs) have been shown to play an essential role in triggering the innate immune response by recognizing pathogen-associated molecular patterns (PAMPs), and in stimulating the activity of host immune cells against several microbial products [3]. A growing number of studies have shown that TLR1-10s are expressed on both human corneal epithelium and fibroblasts [4-6], and that they play an important role in cornea protection and defense against microbial infection [4,6-9].

Glucocorticoids are widely recognized as regulators of adaptive immunity and inflammation and have been extensively used clinically to suppress a large variety of inflammatory and immune responses [10]. Topically, corticosteroids are the most widely used agents and are the standard treatment of nearly every inflammatory disease of the anterior segment [11,12]. The molecular and cellular mechanisms involved in the anti-inflammatory actions of glucocorticoids are now becoming clearer. However, there is no convincing evidence that topical glucocorticoids suppress innate immune responses in the cornea or increase susceptibility to cornea infections.

In this study, we investigated the effects of hydrocortisone on the expression of *TLR2* and *TLR4* in human corneal fibroblast cells (HCFs). The results demonstrated that the functional expression of *TLR2* and *TLR4* is greatly downregulated in HCFs by hydrocortisone. However, these inhibitions can be counteracted after pretreatment with anti-TLR2 and anti-TLR4 monoclonal antibodies. These findings provide evidence for the important role of glucocorticoids on infection keratitis and indicate that the use of topical glucocorticoids may affect the cornea’s innate immunity through TLRs.

## Methods

### Reagents and antibodies

Dulbecco’s Modified Eagle Medium, F12, fetal bovine serum (FBS), and phosphate-buffered saline (PBS) were obtained from Invitrogen-Gibco (New York, NY). All media and cytokines used for cell culture were endotoxin-minimized. Tissue culture dishes and six-well chamber slides were from BD (New York, NY). Hydrocortisone was obtained from Calbiochem (Darmstant, Germany). Affinity-purified, monoclonal, anti-human TLR2, TLR4, and normal mouse immunoglobulin G (IgG) were from eBioscience (San Diego, CA). Paired antibodies for human interleukin-6 (IL-6) and IL-8 enzyme-linked immunosorbent assays (ELISA) were from BD. RNeasy Mini kits were purchased from Qiagen (Valencia, CA) for RNA extraction. RNA PCR kits were from Promega (Fitchburg, WI), and ethidium bromide, DNA molecular size markers, and agarose were from Gene Tech (Shanghai, China). SYBR Green PCR kits were from Applied Biosystems (Foster City, CA).

### Isolation and culture of human corneal fibroblasts

Four human corneas were obtained from the Eye Bank of Wenzhou Medical College (Wenzhou, China). The donors were Chinese males and females ranging in age from 23 to 28 years. After the center of each donor cornea was punched out for corneal transplantation surgery, the remaining rim of the tissue was used for the present experiments. Human material was used in strict accordance with the basic principles of the Declaration of Helsinki. Corneal fibroblasts were prepared and cultured as described previously [13]. Each cornea was digested separately with collagenase to provide a suspension of corneal fibroblasts. The cells from each cornea were cultured independently in DMEM supplemented with 20% FBS in 60 mm dishes until they had achieved ≥90% confluence, then these digested cells were moved from the 60 mm dishes to a 25 cm^2^ culture flask. They were used for the present study after four to six passages. Purity of the corneal fibroblast cultures was judged on the basis of cell morphology and reactivities with antibodies to cytokeratin, as previously described [14]. All the cells were negative for cytokeratin, suggesting that the cultures were not contaminated by epithelial cells.

### Cell challenge

The cells were stimulated with different concentrations of hydrocortisone (1, 10, or 100 μg/ml). For extracting total RNA, the cells were incubated under the stimulation of hydrocortisone for 48 h at 37 °C, and then harvested. For ELISA, the supernatants were incubated under the stimulation of hydrocortisone for 48 h at 37 °C, then collected and stored at −80 °C after centrifugation, until use.

TLR blocking experiments were conducted by incubating HCFs with monoclonal antibodies against TLRs. HCFs were incubated at room temperature with either anti-TLR4, anti-TLR2, both anti-TLR4 and anti-TLR2, or IgG control antibodies for 60 min. Cells were then treated with hyphal fragments for 48 h at 37 °C, and the supernatants were collected in order to evaluate the releases of IL-6 and IL-8.

### Real-time PCR

Total RNA prepared from confluent monolayers of HCFs was used to evaluate the constitutive expression of *TLR2* and *TLR4* mRNA. A negative control (the PCR without a preceding RT step) for each sample was run in order to assess whether there was residual genomic DNA in the DNase-treated samples. Real-time PCR was performed in an ABI PRISM 7500 Sequence Detection System Thermal Cycler (Applied Biosystems). Real-time PCR was performed on a volume of 15 μl containing 1.5 μl (50 ng) of cDNA and 13.5 μl of master mix containing 7.5 μl of mix (SYBR Green PCR Master Mix, Applied Biosystems, UK), 0.75 μl of each primer (10 pmol/l), and 4.5 μl of diethyl pyrocarbonate-treated water. The primers are listed in Table 1. The program was set at 50 °C for 2 min and 95 °C for 10 min, followed by 40 cycles of denaturation at 95 °C for 15 s, and annealing at 60 °C for 60 s. The melting curve was analyzed by elevating the temperature from 60 °C to 95 °C while monitoring ﬂuorescence. SYBR green ﬂuorescence was monitored after each elongation period. Samples were ampliﬁed with GAPDH primers for determination of the initial relative quantity of cDNA in each sample, and then all PCR products were normalized to that amount. Negative controls (without template) were produced for each run.

**Table 1:** Primers for real-time PCR.

**Target gene**	**Locus**	**Forward sequence (5’–3’)**	**Reverse sequence (5’–3’)**	**Amplicon size (bp)**
*VEGF*	NM004716	ACCCCAGGTCAGACGGACAGAA	GGAATCCCCAAAGACCAGCAAT	60
*TLR2*	NM003264	TCTCCCATTTCCGTCTTTTT	GGTCTTGGTGTTCATTATCTTC	125
*TLR4*	NM003266	GAAGCTGGTGGCTGTGGA	TGATGTAGAACCCGCAAG	213
*GAPDH*	NM204305	CCCCACACACATGCACTTACC	TTGCCAAGTTGCCTGTCCTT	100

Samples were amplified in triplicate, averages were calculated, and differences in Ct data were evaluated by Sequence Detection Software V1.3.1 (Applied Biosystems). For data analysis, we used the comparative Ct method (ΔΔ^Ct^ method) with the following formula: Δ^Ct^=Ct (Target, TLR) − Ct (Endo, GAPDH). The comparative ΔΔ^Ct^ calculation involved finding the difference between the Δ^Ct^ of treated cells and the mean value of the Δ^Ct^ from the untreated cells. Fold increase in the expression of specific mRNA in treated cells compared to untreated cells was calculated as 2^-(ΔΔCt)^. Data are expressed as relative quantities (RQs), and differences are shown in the ﬁgures as the expression ratio of the normalized target gene, according to the software results.

### Immunofluorescent staining

HCFs were seeded onto Lab-Tek tissue culture chamber slides without FBS for 24 h. The cells were then washed with Hank's Balanced Salt Solution (Invitrogen-Gibco) and stimulated with 10 μg/ml hydrocortisone for 48 h. The slides were then fixed in 4% paraformaldehyde for 15 min and washed with 10× Tris-buffered saline (TBS) 3 times for 5 min each. Fixed cells were incubated in a blocking buffer of 5% BSA (Proliant) and 0.1% Triton X-100 in PBS for 30 min at room temperature. Cells were then incubated with one or the other of the following dilutions of primary antibodies for 1 h at room temperature: primary mouse anti human TLR2 and TLR4 monoclonal antibodies (20 μg/ml in 5% BSA-PBS) or with mouse IgG (control). The secondary antibodies, conjugated to Cy3, were diluted 1:200 in 5% BSA-PBS and incubated for 1 h at room temperature. Coverslips were washed three times in PBS for 5 min, mounted (Vectashield; Vector Laboratories, Burlingame, CA), and viewed with a fluorescence microscope (Zeiss microscope Imager Z1, Zeiss, Germany). The DNA-intercalating dye, DAPI dihydrochloride, was used to stain nuclei. For the negative control, preimmune mouse serum was substituted for the primary antibody.

### Western blot

Cells challenged with hydrocortisone were lysed in RIPA buffer (150 mM NaCl, 100 mM Tris-HCl [pH 7.5], 1% deoxycholate, 0.1% SDS, 1% Triton X-100, 50 mM NaF, 100 mM sodium pyrophosphate, 3.5 mM sodium orthovanadate, proteinase inhibitor cocktails, and 0.1 mM phenylmethylsulfonyl fluoride [PMSF]). Protein concentration was determined using the bicinchoninic acid (BCA) assay (Micro BCA; Pierce Biotechnology, Rockford, IL). Equal amounts of protein were mixed with SDS-PAGE protein loading buffer and boiled for 5 min. Proteins were separated by sodium dodecyl sulphate–polyacrylamide gel electrophoresis in a Tris/glycine/SDS buffer (25 mM Tris, 250 mM glycine and 0.1% SDS) and electro-blotted onto nitrocellulose transfer membranes. After blocking with 5% nonfat milk for 1 h, membranes were washed three times with TBST for 5 min and incubated overnight with polyclonal antibodies against TLR2, TLR4, and pIκB-α (1:1,000 dilution in 5% nonfat milk) in TBST. GAPDH was used as the control. After washing three times in TBST, membranes were incubated with secondary HRP-conjugated anti-mouse IgG for 1 h. The membranes were again washed with TBST three times, and one time in TBS, for 5 min each. Immune complexes were visualized with an enhanced chemiluminescence reagent (Pierce). Results were quantified by capturing the exposed x-ray film image and using area measurements from image analysis software.

### Enzyme-linked immunosorbent assays

The concentration of IL-6 and IL-8 in the cell culture supernatant fluids was determined by ELISA. The assay was performed according to manufacturer’s instructions. Results from two representative experiments are presented as the mean±SEM of triplicate cytokine measurements.

### Statistical analysis

Data are expressed as mean±SEM of triplicates from experiments repeated three times that yielded similar results. The statistical significance of differences was determined with the non-parametric Wilcoxon test and Student’s t test using SPSS, version 11.5. Differences were considered statistically significant at p<0.05.

## Results

### Modulation of *TLR2* and *TLR4* mRNA expression by hydrocortisone

**Figure 1:**
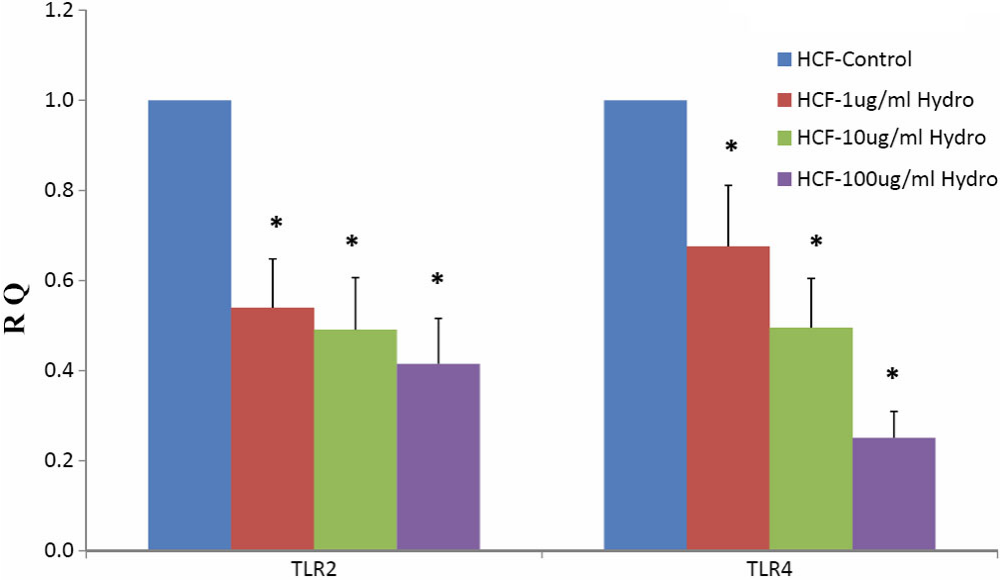
*TLR2* and *TLR4* mRNA expression in hydrocortisone-treated HCF. *TLR2* and *TLR4* mRNA expression in three concentrations of hydrocortisone-treated HCFs, compared with untreated HCFs. Bars represent mean±SEM of 3 independent experiments. The asterisk represents a p value of <0.05, versus the control.

We first wanted to determine if hydrocortisone treatment altered *TLR2* and *TLR4* mRNA expression in cells. The cells were treated with three different concentrations (1, 10, or 100 μg/ml) hydrocortisone at 37 °C for 48 h. The effect of hydrocortisone treatment on *TLR2* and *TLR4* mRNA expression in HCFs is shown in Figure 1. The results indicated that hydrocortisone treatment decreased the TLRs’ mRNA expression in a dose-dependent manner.

### Decreased expression of TLR2 and TLR4 protein following hydrocortisone

**Figure 2:**
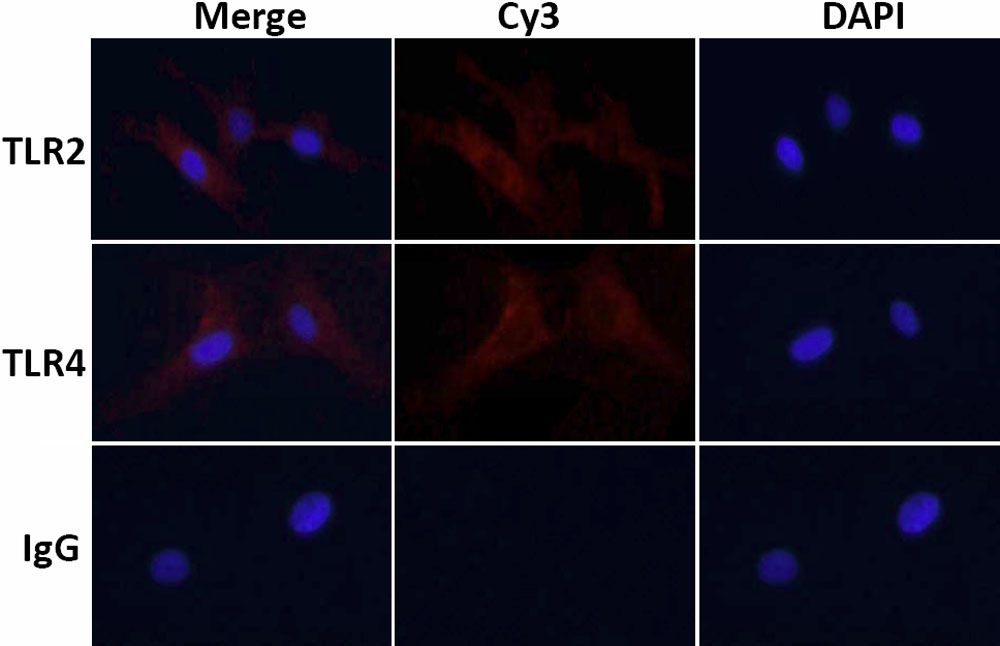
The cells were treated with anti-TLR2 or anti-TLR4 antibodies and stained by Cy3 and DAPI dihydrochloride. There was no immunoreactivity in the negative control (isotype IgG). Merge means overlapping DAPI and Cy3.

**Figure 3:**
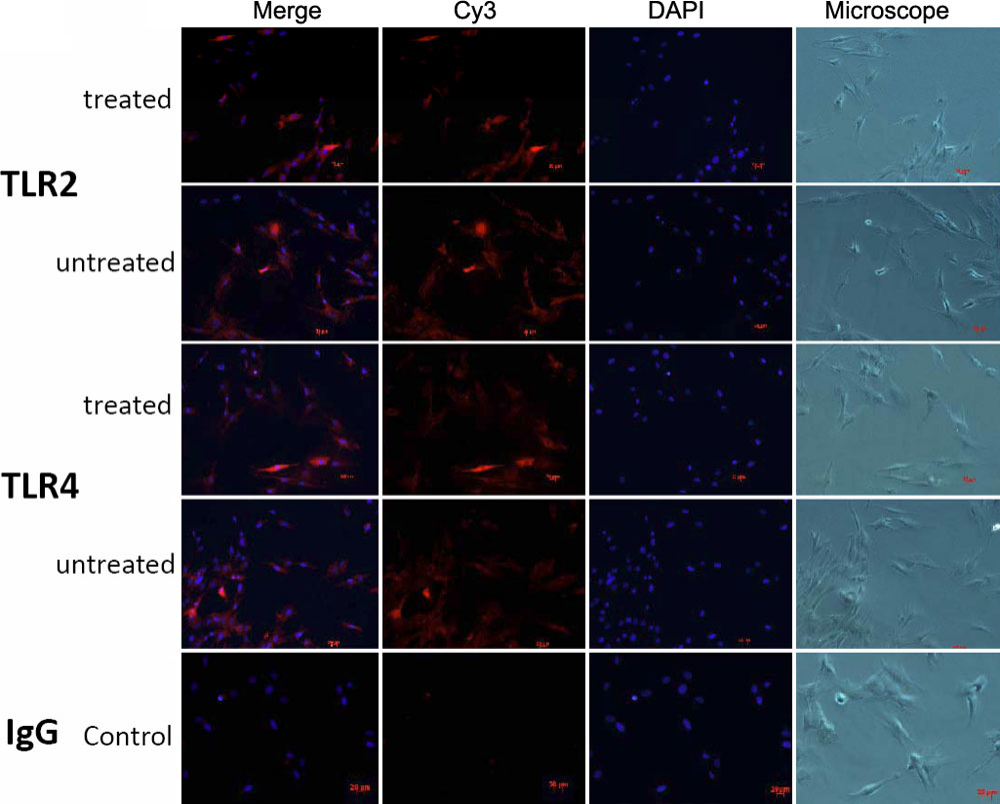
The 10 μg/ml hydrocortisone-stimulated cells treated with anti-TLR2 and anti-TLR4 antibodies and stained by Cy3 and DAPI dihydrochloride. There was no immunoreactivity in the negative control (isotype IgG). Merge means overlapping DAPI and Cy3.

The results of immunofluorescence staining revealed moderate TLR2 and TLR4 reactivity in untreated HCFs (Figure 2). The staining intensity of these antigens was slightly inhibited after treatment with hydrocortisone at 10 μg/ml concentration (Figure 3).

**Figure 4:**
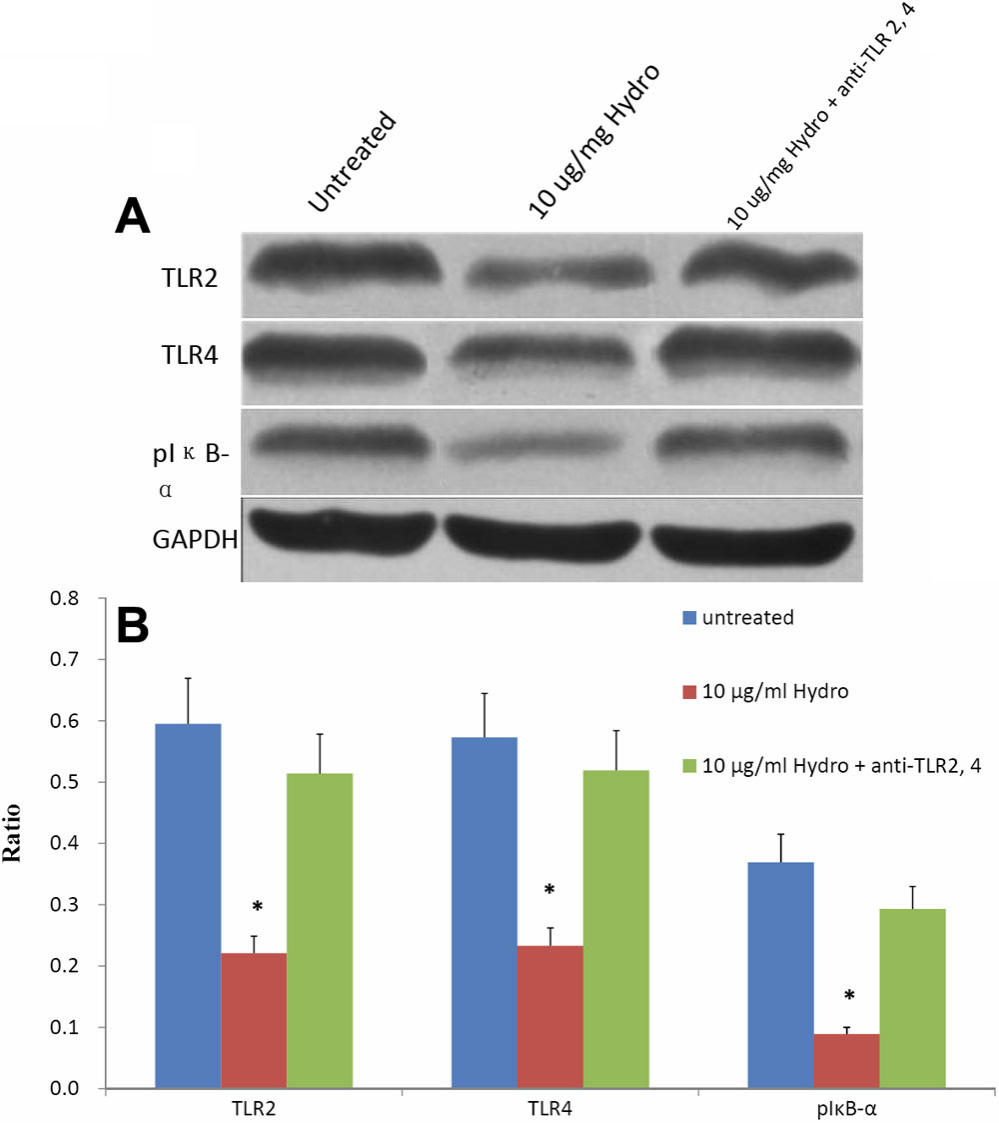
The expression of TLR2, TLR4, and pIκB-α under stimulation. **A**: Western blot analyses detect the expression of TLR2, TLR4, and pIκB-α at the protein level in HCFs under stimulation of 10 μg/ml hydrocortisone or pretreated with anti-TLR2 and anti-TLR4 monoclonal antibodies. Equal amounts of proteins were loaded. **B**: Column diagrams and bars represent mean±SEM for the scanned immunoblots (the ratio of TLRs to GAPDH. The results are representative of three independent experiments. In the image, 10-Hy indicates 10 μg/ml hydrocortisone. The asterisk represents a p value of <0.05, versus untreated HCFs.

The expression of TLR2 and TLR4 was also confirmed by western blot analysis. We next evaluated the protein expression in HCFs of TLR 2 and TLR4 under hydrocortisone (10 μg/ml) treatment. As evidenced in Figure 4A,B, the expression of TLR2 and TLR4 protein can be downregulated in HCFs at the protein level using western blot with the cellular protein GAPDH as the standard. The expression of TLR2 and TLR4 proteins following treatment with hydrocortisone was counteracted after pretreatment with anti-TLR2 and anti-TLR4 monoclonal antibodies.

### Detection of pIκB-α protein on human corneal fibroblasts by western blot

The results of western blot analysis demonstrated decreased expression of pIκB-α following treatment with hydrocortisone. The expression of pIκB-α following treatment with hydrocortisone was counteracted after pretreatment with anti-TLR2 and anti-TLR4 monoclonal antibodies (Figure 4A,B).

### Pretreatment with specific TLR2 and TLR4 monoclonal antibodies counteracted the hydrocortisone-inhibited release of IL-6 and IL-8

**Figure 5:**
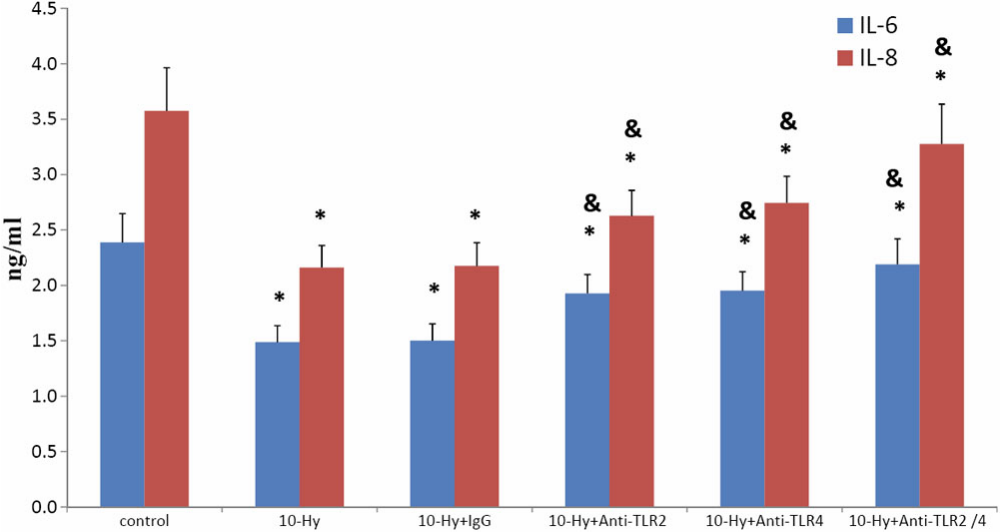
The results of ELISA showed the release of IL-6 and IL-8 from HCFs under different stimulation. Data are the mean±SEM of triplicates from an experiment that was repeated three times with similar results. The asterisk indicates p<0.05 versus the control, and p<0.05 versus 10 μg/ml hydrocortisone.

**Figure 6:**
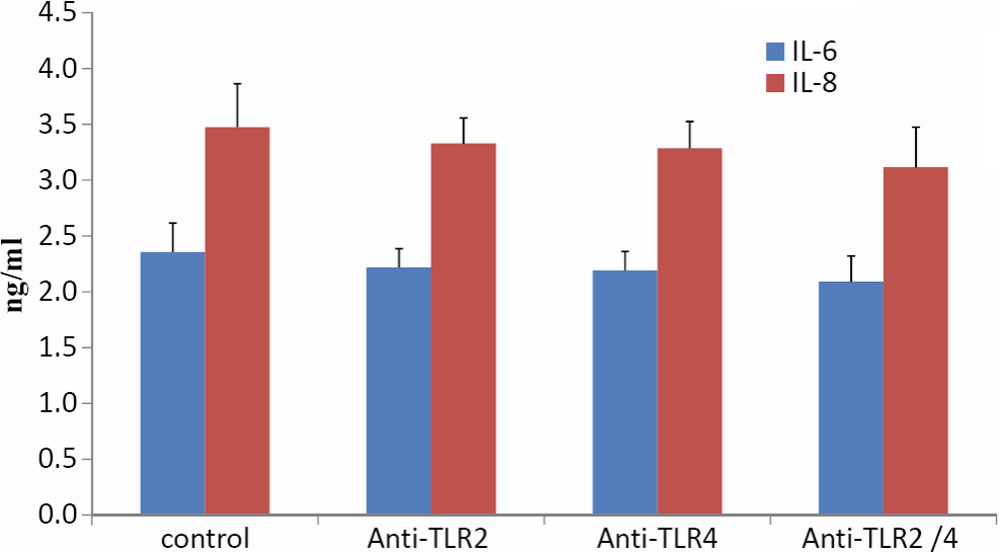
The results of ELISA showed the release of IL-6 and IL-8 from HCFs only pretreated with anti-TLR2 and anti-TLR4 monoclonal antibodies. Data are the mean±SEM of triplicates from an experiment that was repeated three times with similar results. The results show that incubation with anti-TLR2 and/or anti-TLR4 mAb had little effect on the production of IL-6 and IL-8.

HCFs were incubated at room temperature either with anti-TLR4, anti-TLR2, both anti-TLR4 and anti-TLR2, or IgG control antibodies for 1 h. Cells were then treated with hydrocortisone (10 μg/ml) for 48 h at 37 °C; the supernatants were collected to evaluate the release of IL-6 and IL-8. The results of ELISA showed that pretreatment of HCFs with anti-TLR2 and/or anti-TLR4 inhibited the production of IL-6 and IL-8 following exposure to hydrocortisone. Figure 4 shows that anti-TLR2 and anti-TLR4 pretreatment reverses hydrocortisone suppression of IL-6 and IL-8 expression. In contrast, an isotype-matched control, Ab, had no effect on the release of IL-6 and IL-8 (Figure 5). Maximal upregulation was observed in HCF cells treated with antibodies against both TLR2 and TLR4. In comparison, incubation with anti-TLR2 and/or anti-TLR4 mAb had little effect on the production of IL-6 and IL-8 without following exposure to hydrocortisone (Figure 6).

## Discussion

Glucocorticoid potently suppresses immunity and is commonly used in the treatment of a wide variety of immune and inflammatory diseases [15]. Currently, topical corticosteroids are the main choice of anti-inflammatory agents for the management of most ocular surface immune-mediated diseases [11,14,16]. Our previous report indicated that hydrocortisone treatment increases mRNA expression of *TLR2* and *TLR4* in human corneal epithelial cell lines (HCEC) [17]. The results indicate that hydrocortisone may increase the innate immunity of the HCEC through TLRs. Although the expression of TLR-specific mRNAs of fibroblasts has previously been studied [5,18,19], there are no extensive reports on the expression and function of TLRs in HCFs. In this work, we have shown that *TLR2* and *TLR4* mRNA expression can be downregulated by hydrocortisone in a dose-dependent manner. Western blotting also showed that the protein expression of TLR2 and TLR4 was also downregulated following pretreatment with hydrocortisone in HCFs. These results suggest that hydrocortisone may suppress the innate immunity of HCFs by inhibiting the expression of TLRs.

Corticosteroids inhibit inflammation through various pathways. For instance, corticosteroid-induced MAPK phosphatase 1 dephosphorylates and inactivates Jun N-terminal kinase, thereby inhibiting c-Jun-mediated transcription [15]. Corticosteroid-glucocorticoid receptor complex also interacts with NF-κB to block its transcription activity [15]. As we know, recognition of pathogen-associated molecular patterns via TLRs can lead to translocation of the NF-κB, with consequent upregulation of proinflammatory cytokines, co-stimulatory molecules, and chemokines, such as TNF-α, IL-6, IL-8, IL-18, and monocyte chemotactic protein-1 (MCP) [20,21]. Our results show that the expression of TLR2 and TLR4 was markedly inhibited by hydrocortisone in both mRNA and at the protein level. At the same time, the release of IL-6 and IL-8 can also be inhibited by hydrocortisone. The results also agree with those of Lu et al. [22], who have shown that dexamethasone, inhibited, also in a dose-dependent manner, the release of IL-8 and MCP-1 from human corneal fibroblasts induced by TNF-α or IL-1β. The TLR family of receptors links the extracellular compartment, where contact and recognition of PAMPs occur, and the intracellular compartment, where signaling cascades leading to cellular responses are initiated. Li and his associates [23] have recently reported that glucocorticoid enhanced the expression of TLR-2 by inhibiting the negative effect of p38 MAPK kinase, by increasing the expression of the phosphatase mitogen-activated protein kinase phosphatase-1 (MKP)-1. Silverstein et al. [24] reported that TLR2 is involved in glucocorticoid’s protective efficacy against Gram-positive and Gram-negative sepsis in experimental bacterial sepsis. Anti-TLRs antibodies have been long used to study the function of TLRs. Many reports have suggested anti-TLR antibodies can inhibit the function of TLRs [17,25,26]. However, there is no report about the impact of the use of glucocorticoids on innate immunity in the absence of stimuli such as LPS, poly (I: C), and so forth.

In order to further determine if the role of glucocorticoids may relate to TLRs, the cultured HCFs were pretreated with specific TLR2 and TLR4 monoclonal antibodies to study the expression of TLR2, TLR4, IL-6, and IL-8. We found that the expression of TLR2 and TLR4, and the release of IL-6 and IL-8, which was inhibited by hydrocortisone, can be partly counteracted by anti-TLR2 and anti-TLR4 monoclonal antibodies. For detailed analysis of the specific contribution of TLRs, we investigated NF-κB activation in hydrocortisone-treated HCFs. Because phosphorylation of IκB-α at Ser32 is essential for the release of active NF-κB, phosphorylation at this site is an excellent marker of NF-κB activation. Western blot analysis revealed that pIkB-α activation was downregulated in hydrocortisone-treated HCFs, but the expression can be partly counteracted by anti-TLR2 and anti-TLR4 monoclonal antibodies. These results further indicate that hydrocortisone may suppress the innate immunity of HCFs through TLR2 and TLR4. Yet why the glucocorticoids can act on the TLRs is still unknown, because no ligands were used in this study. We speculate that the possible mechanism may be the following. 1) Glucocorticoid blocks the production of many mediators of immune and inflammatory response, such as cytokines, chemokines, and cell adhesion molecules. As a consequence, glucocorticoids may act on the TLRs through cytokines and chemokines. 2) Glucocorticoid had direct effects on TLRs because there may exist cross-action between glucocorticoid receptors and TLRs. 3) Glucocorticoid had direct effects on TLRs as an unspecific ligand of TLRs. This study provides insight into the mechanism of the action of glucocorticoid in the treatment of corneal disease. However, the molecular mechanisms of glucocorticoid’s direct or indirect effects on TLR2 and TLR4 expression still need further investigation. Although the cornea is highly resistant to infections under normal conditions, sight-threatening microbial infections may occur when the corneal integrity is breached by trauma or by wear from a contact lens. Therefore, the underlying mechanisms that regulate corneal fibroblast cell activation are important in the development of infectious keratitis. As we know, steroid application has been used to promote bacterial, fungal, viral, and acanthamoebic cornea infections of animal models [22-30]. Thus, our results suggest that hydrocortisone’s suppression of the innate immunity of HCFs through TLRs may explain why hydrocortisone can promote opportunistic cornea infection. Our results indicated the action of hydrocortisone on HCFs was related to TLR2 and TLR4, but whether other TLRs also play a crucial role requires further investigation.

## References

[1] Thakur A Willcox MD Cytokine and lipid inflammatory mediator profile of human tears during contact lens associated inflammatory diseases Exp Eye Res 1998 67 9 19 9702174 10.1006/exer.1998.0480

[2] Spandau UH Toksoy A Verhaart S Gillitzer R Kruse FE High expression of chemokines Gro-[alpha] (CXCL-1), IL-8 (CXCL-8), and MCP-1 (CCL-2) in inflamed human corneas in vivo. Arch Ophthalmol 2003 121 825 31 12796254 10.1001/archopht.121.6.825

[3] Medzhitov R Janeway CA Jr Innate immunity: the virtues of a nonclonal system of recognition Cell 1997 91 295 8 9363937 10.1016/s0092-8674(00)80412-2

[4] Ueta M Hamuro J Kiyono H Kinoshita S Triggering of TLR3 by polyI:C in human corneal epithelial cells to induce inflammatory cytokines. Biochem Biophys Res Commun 2005 331 285 94 15845391 10.1016/j.bbrc.2005.02.196

[5] Kumar A Yu FS Toll-Like Receptors and Corneal Innate Immunity Curr Mol Med 2006 6 327 37 16712478 10.2174/156652406776894572 PMC2666391

[6] Kumar A Zhang J Yu FS Innate immune response of corneal epithelial cells to Staphylococcus aureus infection: role of peptidoglycan in stimulating proinflammatory cytokine secretion Invest Ophthalmol Vis Sci 2004 45 3513 22 15452057 10.1167/iovs.04-0467 PMC2666393

[7] Zhang J Xu K Ambati B Yu FS Toll-like receptor 5-mediated corneal epithelial inflammatory responses to Pseudomonas aeruginosa flagellin. Invest Ophthalmol Vis Sci 2003 44 4247 54 14507868 10.1167/iovs.03-0219

[8] Song PI Abraham TA Park Y Zivony AS Harten B Edelhauser HF Ward SL Armstrong CA Ansel JC The expression of functional LPS receptor proteins CD14 and toll-like receptor 4 in human corneal cells. Invest Ophthalmol Vis Sci 2001 42 2867 77 11687531

[9] Ueta M Nochi T Jang MH Park EJ Igarashi O Hino A Kawasaki S Shikina T Hiroi T Kinoshita S Kiyono H Intracellularly expressed TLR2s and TLR4s contribution to an immunosilent environment at the ocular mucosal epithelium. J Immunol 2004 173 3337 47 15322197 10.4049/jimmunol.173.5.3337

[10] De Bosscher K Vanden Berghe W Haegeman G The Interplay between the Glucocorticoid Receptor and Nuclear Factor-κB or Activator Protein-1: Molecular Mechanisms for Gene Repression Endocr Rev 2003 24 488 522 12920152 10.1210/er.2002-0006

[11] Leibowitz HM Kupferman A Antiinflammatory medications Int Ophthalmol Clin 1980 20 117 34 6998894 10.1097/00004397-198002030-00012

[12] De Bosscher K Vanden Berghe W Haegeman G Mechanisms of anti-inflammatory action and of immunosuppression by glucocorticoids: negative interference of activated glucocorticoid receptor with transcription factors J Neuroimmunol 2000 109 16 22 10969176 10.1016/s0165-5728(00)00297-6

[13] Kumagai N Fukuda K Nishida T Synergistic effect of TNF-α and IL-4 on the expression of thymus- and activation-regulated chemokine in human corneal fibroblasts Biochem Biophys Res Commun 2000 279 1 5 11112408 10.1006/bbrc.2000.3890

[14] Foster CS. Immunologic disorders of the conjunctiva, cornea, and sclera. In: Albert DM, Jakobiec FA, editors. Principles and Practice of Ophthalmology. 2nd ed. Philadelphia: W.B. Saunders; 2000. p.823-29.

[15] Rhen T Cidlowski JA Antiinflammatory action of glucocorticoids—new mechanisms for old drugs. N Engl J Med 2005 353 1711 23 16236742 10.1056/NEJMra050541

[16] Dana MR Qian Y Hamrah P Twenty-five year panorama of corneal immunology Cornea 2000 19 625 43 11009315 10.1097/00003226-200009000-00008

[17] Jin X Qin Q Tu L Zhou X Lin Y Qu J Toll-like receptors (TLRs) expression and function in response to inactivate hyphae of Fusarium solani in immortalized human corneal epithelial cells. Mol Vis 2007 13 1953 61 17982419 PMC2185515

[18] Kurt-Jones EA Sandor F Ortiz Y Bowen GN Counter SL Wang TC Finberg RW Use of murine embryonic fibroblasts to define Toll-like receptor activation and specificity. J Endotoxin Res 2004 10 419 24 15588425 10.1179/096805104225006516

[19] Otte J-M Rosenberg IM Podolaky DK Intestinal Myofibroblasts in Innate Immune Responses of the Intestine Gastroenterology 2003 124 1866 78 12806620 10.1016/s0016-5085(03)00403-7

[20] Barton GM Medzhitov R Toll-Like receptor signaling pathways Science 2003 300 1524 5 12791976 10.1126/science.1085536

[21] Luster AD The role of chemokines in linking innate and adaptive immunity Curr Opin Immunol 2002 14 129 35 11790543 10.1016/s0952-7915(01)00308-9

[22] Lu Y Fukuda K Nakamura Y Kimura K Kumagai N Nishida T Inhibitory Effect of Triptolide on Chemokine Expression Induced by Proinflammatory Cytokines in Human Corneal Fibroblasts. Invest Ophthalmol Vis Sci 2005 46 2346 52 15980221 10.1167/iovs.05-0010

[23] Imasato A Desbois-Mouthon C Han J Kai H Cato AC Akira S Li JD Inhibition of p38 MAPK by glucocorticoids via induction of MAP kinase phosphatase-1 enhance nontypeable Haemophilus influenzae- induced expression of Toll-like receptor 2. J Biol Chem 2002 277 47444 50 12356755 10.1074/jbc.M208140200

[24] Silverstein R Johnson DC Endogenous versus exogenous glucocorticoid responses to experimental bacterial sepsis. J Leukoc Biol 2003 73 417 27 12660216 10.1189/jlb.0702379

[25] Hertz CJ Kiertscher SM Godowski PJ Bouis DA Norgard MV Roth MD Modlin RL Microbial lipopeptides stimulate dendritic cell maturation via Toll-like receptor 2. J Immunol 2001 166 2444 50 11160304 10.4049/jimmunol.166.4.2444

[26] Melkamu T Squillace D Kita H O'Grady SM Regulation of TLR2 expression and function in human airway epithelial cells. J Membr Biol 2009 229 101 13 19513781 10.1007/s00232-009-9175-3

[27] Lee EJ Truong TN Mendoza MN Fleiszig SM A comparison of invasive and cytotoxic Pseudomonas aeruginosa strain-induced corneal disease responses to therapeutics. Curr Eye Res 2003 27 289 99 14562165 10.1076/ceyr.27.5.289.17220

[28] Wu TG Wilhelmus KR Mitchell BM Experimental Keratomycosis in a Mouse Model. Invest Ophthalmol Vis Sci 2003 44 210 6 12506077 10.1167/iovs.02-0446

[29] Romanowski EG Yates KA Gordon YJ Topical Corticosteroids of Limited Potency Promote Adenovirus Replication in the Ad5/NZW Rabbit Ocular Model. Cornea 2002 21 289 91 11917178 10.1097/00003226-200204000-00010

[30] McClellan K Howard K Niederkorn JY Alizadeh H Effect of Steroids on Acanthamoeba Cysts and Trophozoites. Invest Ophthalmol Vis Sci 2001 42 2885 93 11687533

